# On QSAR-based cardiotoxicity modeling with the expressiveness-enhanced graph learning model and dual-threshold scheme

**DOI:** 10.3389/fphys.2023.1156286

**Published:** 2023-05-09

**Authors:** Huijia Wang, Guangxian Zhu, Leighton T. Izu, Ye Chen-Izu, Naoaki Ono, MD Altaf-Ul-Amin, Shigehiko Kanaya, Ming Huang

**Affiliations:** ^1^ Graduate School of Science and Technology, Nara Institute of Science and Technology, Ikoma, Japan; ^2^ Department of Pharmacology, University of California, Davis, CA, United States; ^3^ Department of Biomedical Engineering, University of California, Davis, CA, United States; ^4^ Data Science Center, Nara Institute of Science and Technology, Ikoma, Japan

**Keywords:** hERG, cardiotoxicity, graph transformer neural network, meta-path, dual-threshold

## Abstract

**Introduction:** Given the direct association with malignant ventricular arrhythmias, cardiotoxicity is a major concern in drug design. In the past decades, computational models based on the quantitative structure–activity relationship have been proposed to screen out cardiotoxic compounds and have shown promising results. The combination of molecular fingerprint and the machine learning model shows stable performance for a wide spectrum of problems; however, not long after the advent of the graph neural network (GNN) deep learning model and its variant (e.g., graph transformer), it has become the principal way of quantitative structure–activity relationship-based modeling for its high flexibility in feature extraction and decision rule generation. Despite all these progresses, the expressiveness (the ability of a program to identify non-isomorphic graph structures) of the GNN model is bounded by the WL isomorphism test, and a suitable thresholding scheme that relates directly to the sensitivity and credibility of a model is still an open question.

**Methods:** In this research, we further improved the expressiveness of the GNN model by introducing the substructure-aware bias by the graph subgraph transformer network model. Moreover, to propose the most appropriate thresholding scheme, a comprehensive comparison of the thresholding schemes was conducted.

**Results:** Based on these improvements, the best model attains performance with 90.4% precision, 90.4% recall, and 90.5% F1-score with a dual-threshold scheme (active: <1μM ; non-active: >30μM ). The improved pipeline (graph subgraph transformer network model and thresholding scheme) also shows its advantages in terms of the activity cliff problem and model interpretability.

## 1 Introduction

It is known that cardiotoxicity is a staple step for healthcare in the cardiovascular drug design because cardiotoxicity leads to electrophysiological dysfunction of the heart or muscle damage (Hayat (2017); Albini et al. (2010)). Therefore, cardiotoxicity is the main reason for the failure of clinical candidates or post-market withdrawal (Klon, 2010). Genetically pharmacological studies evidence the causalities to cardiotoxicity is the human ether-à-go-go-related gene (hERG) ion channel protein expressed in the cardiomyocyte (Comollo et al. (2022); Warmke and Ganetzky (1994)). The hERG gene encodes a voltage-gated potassium channel, which is a key component in the formation of the cardiac action potential by activating the delayed rectifier potassium current rapidly (Cavalluzzi et al., 2020). The extended pockets of the hERG channel are sensitive to a wide range of drugs (Wang and MacKinnon, 2017): the binding to the hERG may result in blockage of the potassium ion channel, which delays the repolarization of the action potential across the cell membrane, potentially leading to prolongation of the QT interval in the electrocardiogram (ECG) (Kratz et al. (2017); Curran et al. (1995)). QT prolongation leads to a potentially lethal form of ventricular tachycardia associated with decreased blood pressure and fainting (Viskin (1999); Aerssens and Paulussen (2005)). Despite the strictly controlled process that has been taken for drug development, it has been confirmed that many types of drugs, including antibiotics, antimalarials, gastroprokinetics, antiarrhythmics, and antipsychotics, bind with the hERG (Ma et al., 2022). Therefore, the assessment of hERG-related cardiotoxicity now is recognized as a common practice in the preclinical stage of drug discovery (Sanguinetti and Tristani-Firouzi, 2006).

Determining hERG-related cardiotoxicity in a medical manner has two approaches: non-electrophysiological and electrophysiological methodologies (Yu et al., 2016a). Considering that the effectiveness of non-electrophysiological metrics is still an open question (e.g., radioligand binding assays and rubidium efflux assays), electrophysiological methods predominate (e.g., the well-known patch clamp) (Villoutreix and Taboureau, 2015). Electrophysiological methods involve measurements of voltage changes, and voltage states can significantly alter hERG *IC*_50_ values, which may not be reflected in non-electrophysiology assays (Jing et al. (2015); Scanziani and Häusser (2009); Park et al. (2013)). However, electrophysiological experiments are time consuming with low throughput, and the procedure usually relies on highly trained practitioners (Yu et al. (2016b); Kornreich (2007)). Albeit with the rapid progress in instrumental automation, the advanced throughput is far from satisfactory given tens of thousands of candidates (Wang and MacKinnon, 2017).

Quantitative structure–activity relationship (QSAR) is a general concept for computational models, which is proposed based on the presumption that similar chemical structures give birth to similar bioactivities (Harkati et al., 2016). Conventionally, the computational model can be divided into two consecutive but independent steps: chemical representation and structure–bioactivity association (Butler et al., 2018). Regarding chemical representation, molecular fingerprint, which is a predefined way to characterize molecular structures by annotating the chemical substructures, is pervasively used. Amongst a variety of fingerprints, the extended connectivity fingerprint (ECFP) is an efficient way for characterization (Morgan, 1965). The ECFP extracts substructures iteratively as the inclusion radius increases and assigns an identifier to each unique substructure. Of note, in the final fingerprint, the order of identifiers is rearranged, and the connectivity between substructures is not included (Rogers and Hahn, 2010). In the second step, machine learning algorithms, such as support vector machine and partial least squares regression, are used to associate the representation and the targeted properties (Rathman et al., 2018).

The utilization of graph neural networks (GNNs) in molecular representation generation has been gaining increasing attention in the field of computational chemistry as a promising approach. Unlike traditional, modular computational models, GNNs offer a more flexible and problem-oriented end-to-end framework (Reiser et al., 2022). This flexibility is a result of the GNNs’ ability to effectively incorporate the interdependencies between the various steps involved in molecular representation generation, as opposed to the mutually independent steps of conventional models (Zhou et al., 2020). The adoption of GNNs as a framework for molecular representation generation can, thus, provide a more holistic and efficient approach to the analysis of chemical systems (Reiser et al.,(2022). In GNN models, graphs, as a fundamental data structure, model a set of entities (nodes) and their interconnections (edges). In this context, molecules can be conceptualized as graphs, with atoms serving as the nodes and chemical bonds serving as the edges. The application of GNNs to molecular graphs has the potential to generate more robust molecular fingerprints, thus enhancing the prediction of molecular properties (Duvenaud et al., 2015). The capability of the GNN and its variant has been validated in the studies by Koge et al. (2021) and Miyazaki et al. (2020).

Even though the molecular structure of the hERG channel has been resolved recently, the binding of chemical compounds to the hERG molecule is still difficult to predict due to the high flexibility in the structure. Hence, ligand-based in-silico modeling is still a highly attractive approach in cardiotoxicity prediction (Chen et al., 2018). Doddareddy et al. (2010) collected a dataset of 2,644 compounds and used them to build an SVM classification model based on ECFP for hERG blockers. Chavan et al. (2016) tried to use a consensus model based on the k-nearest neighbor model (KNN) input with eight types of fingerprints to predict the cardiotoxicity for a dataset of 172 compounds, and a careful comparison of different models, including random forest and SVM, was conducted by Delre et al. (2022) to extract the best pipeline of cardiotoxicity modeling. Cai et al. (2019) constructed a multi-task deep neural network input with Mol2vec feature vectors and MOE descriptors to predict the cardiotoxicity for a total of 7,889 compounds.

Before devising a computational model of cardiotoxicity, insight into this problem, i.e., domain knowledge, should be indicated and addressed in the modeling process. Molecular activity cliff (AC) refers to pairs of molecules with similar structures but greatly different bioactivity potency (Ayed, 2022). It is a common phenomenon that could be misleading for machine learning models based on the QSAR principle (Van Tilborg et al., 2022). Consequently, the problem of “*how to build a model to find the key feature from massive redundant data in active cliff molecules?”* is actually problem oriented. Based on this idea, the match molecular pair (MMP) dataset (Park et al., 2022) and the subsequent MMP fingerprint (Tamura et al., 2021) are erected to facilitate AC identification. The MMP fingerprint represents a molecule by taking in its activity cliff counterpart, that is, to encode the shared core (scaffold), as well as the distinction and resemblance of the two substituents in a binary vector of a variable length (Tamura et al., 2021). In a study that focuses on the detection of AC detection, this relative distance is plausible and efficient. However, in a more comprehensive problem where the AC is only one of the influential factors of a model, a self-context-aware encoding of the core and the branches is desirable. Self-context-aware means that the relations between the core and the branches of a molecule are embedded in some way. It is different from global-structure-aware, where only the relation between the core and a specific branch is considered.

Another issue is the unambiguous labeling, which is a non-trivial step in the process of computational modeling (Villoutreix and Taboureau, 2015). Esposito et al. and Cai et al. tried to use automated procedures to select the optimal threshold (Esposito et al. (2021); Cai et al. (2019)). However, given that the optimal threshold should be biomedically sound as well, there is no gold standard for the threshold of cardiotoxicity as of now, although 1, 10, and 30 *μM* are the most commonly used for thresholding (Konda et al. (2019); Zhang et al. (2016); Kim and Nam (2020); Ryu et al. (2020); Zhang et al. (2022)). Recently, some studies have used a dual-threshold approach in modeling (Creanza et al., 2021). A multi-task DNN model was constructed by Cai et al. to investigate different inactive thresholds (10, 20, 40, 60, 80, and 100 *μM*); the accuracy was 0.81, 0.82, 0.89, 0.90, 0.93, and 0.91, respectively Cai et al. (2019). Even though Cai’s model obtained an accuracy of 0.93 with the 80 *μM* threshold, the resultant dataset was unbalanced (3,485 positives vs. 469 negatives), and the class-wise performances for the blocker and non-blocker differed greatly (0.99 vs. 0.62).

Stemming from the aforementioned considerations, the presented work tries to introduce a subgraph bias for message passing and aggregation based on domain knowledge about cardiotoxicity. By passing the message from the neighboring nodes with the same and different kinds of subgraphs (predefined meta-paths), the new framework is expected to improve the expressiveness of the conventional GNN model. In this manner, heterogeneity that has been abolished by the simplification of homogeneity of the GNN model can also be partially recovered. The domain knowledge-inspired method uses the predefined subgraphs to express the heterogeneity of chemical structure and implicitly injects the structural information into the nodes by introducing the learnable weights for the subgraphs. By introducing the problem-wise subgraph bias into the model, it is believed that the framework could be applied to a broad spectrum of problems that need to incorporate heterogeneity into the model. The contribution of this study is summarized as follows:

• We proposed a new graph neural model whose expressiveness is lifted by introducing the cardiotoxicity-specific subgraph aware bias.
• We determined a dual-threshold mechanism for computational modeling based on a comprehensive validation (a dedicated hERG database and 50 FDA-approved drugs).

The aforementioned improved pipeline is expected to be more robust against the activity cliff and to further improve the performance of the model.

## 2 Materials and methods

### 2.1 Data collection

The dataset *hERG*-*DB* is extracted from the hEMBL bioactivity database (Creanza et al., 2021) and used as the main training and testing datasets. The supplementary material provides details regarding the selection method. For sparsity consideration of adjacency matrices, we selected molecules with less than 45 non-hydrogen atoms from the *hERG*-*DB* for training and testing, which covers 99.0% of the molecules in the entire dataset. As a result, the dataset in our study contains 8,253 molecules. Furthermore, 50 FDA-approved drugs compiled by Zhang et al. (2022) were used to examine the proposed method’s performance and finalize the optimized thresholding scheme.

As explained in *Introduction*, the setting of a threshold for computational modeling is still controversial. Therefore, single- and dual-threshold schemes with different values (Table 1) are applied to the dataset for a comprehensive comparison. Regarding the single-threshold scheme, in addition to the commonly used 1 and 10 *μM*, we consider using 20 *μM*. In the case of the dual-threshold scheme, the values that define the blockers (active threshold) and the decoys (inactive threshold) are different. For example, the dual threshold “ ≤1μM,>10μM ” means that molecules with *IC*_50_ values less than 1 *μM* are viewed as blockers, while those with *IC*_50_ values higher than 10 *μM* are viewed as non-blockers. The ones with *IC*_50_ values between 1 and 10 *μM* are removed from the dataset. The dual threshold is also denoted as [XX, YY] *μM* hereafter, where XX represents the blocker threshold and YY represents the non-blocker threshold. In determining the dual threshold, the active threshold is relatively well defined at 1 *μM* and, thus, fixed throughout the study. A comparison is made between the inactive thresholds of 10, 20, 30, and 40 *μM*, as indicated in Table 1. Additionally, the balanced version is created by randomly selecting non-blockers and maintaining the same sample number as the dual-threshold datasets.

**TABLE 1:** Datasets of different thresholds.

Usage	Range	Class		Sample no.	All
**One threshold**	≤1μM,>1μM	>1μM	Non-blocker	6,958	8,253
		≤1μM	Blocker	1,295	
	≤10μM,>10μM	>10μM	Non-blocker	3,722	8,253
		≤10μM	Blocker	4,531	
	≤20μM,>20μM	>20μM	Non-blocker	2,871	8,253
		≤20μM	Blocker	5,283	
**Dual threshold/imbalanced**	≤1μM,>1μM	>1μM	Non-blocker	6,958	8,253
		≤1μM	Blocker	1,295	
	≤1μM,>10μM	>10μM	Non-blocker	3,722	5,017
		≤1μM	Blocker	1,295	
	≤1μM,>20μM	>20μM	Non-blocker	2,871	4,166
		≤1μM	Blocker	1,295	
	≤1μM,>30μM	>30μM	Non-blocker	2,052	3,347
		≤1μM	Blocker	1,295	
	≤1μM,>40μM	>40μM	Non-blocker	1,358	2,653
		≤1μM	Blocker	1,295	
**Balanced**	≤1μM,>1μM	>1μM	Non-blocker	1,295	2,590
		≤1μM	Blocker	1,295	
	≤1μM,>10μM	>10μM	Non-blocker	1,295	2,590
		≤1μM	Blocker	1,295	
	≤1μM,>20μM	>20μM	Non-blocker	1,295	2,590
		≤1μM	Blocker	1,295	
	≤1μM,>30μM	>30μM	Non-blocker	1,295	2,590
		≤1μM	Blocker	1,295	
	≤1μM,>40μM	>40μM	Non-blocker	1,295	2,590
		≤1μM	Blocker	1,295	

### 2.2 Graph subgraph transformer network model

#### 2.2.1 Molecular graph representation

To represent the properties of molecules in a computational format, that is, to define a molecule with *N* atoms as two main components, the adjacency matrix and feature matrix, the adjacency matrix *A*, which is defined by $A\in R^{N\times N}$ , represents the topological information of the molecule, such as the connectivity of atoms. The *D*-dimension feature matrix *X*, which is defined by $X\in R^{N\times D}$ , stores information about the atomic features of the molecule, such as atomic properties and location in relation to the topological information represented in the adjacency matrix. This information can then be used for computational analysis and simulations of molecular systems.

#### 2.2.2 Subgraph bias expressiveness enhancement

It has been shown that the graph neural networks based on the message passing and aggregation scheme are the neural approaches of the 1st-order Weisfeiler–Leman algorithm (1-WL) (Morris et al., 2021). Given that the 1-WL fails at distinguishing some basic non-isomorphic graph structures, improvements/amendments, such as the GNN for higher-order WL or additional structure-aware information, have been tried to make the GNN universal (refer to the universal approximation theorem of neural network) (Bouritsas et al., 2020).

On the other hand, it is well known that the simplification from a heterogeneous graph into the homogeneous one, which is the underlying requirement of the conventional GNN model, abolishes the relational information that is important for chemical structure. In this paper, we propose a domain knowledge-inspired technique that uses the predefined substructure (subgraph) to 1) express the heterogeneity of chemical structure and 2) implicitly inject the structural information into the nodes by introducing the learnable weights for the predefined subgraphs.

Specifically, subgraph bias to enhance the expressiveness of a GNN model is introduced based on the domain knowledge in cardiotoxicity screening. The chemical core, the Murcko scaffold, is extracted as a special subgraph. Alongside the whole molecule being another subgraph, context-aware information can be encoded. In addition, the aromatic rings and the heteroatoms (nitrogen, oxygen, and sulfur atoms) are also extracted as independent subgraphs because their interactions with residues are reported responsible for hERG blockage (Creanza et al. (2021); Moorthy et al. (2021); Kim and Nam (2020)).

#### 2.2.3 Heterogeneous decomposition mapping

In order to integrate the heterogeneous information presented by the subgraphs defined above, we propose a mapping function that decomposes the adjacency matrix *A* into *T* sub-adjacency matrices (subgraphs) denoted by *G*_*i*_, *i* ∈ {1, *…*, *T*}. Here, *i* represents the index of the subgraphs, ranging from 1 to *T*, and *T* is the number of subgraphs. To facilitate this integration of subgraphs, adjacency matrix *A* is transformed into a *T*-dimensional tensor $M\in R^{T\times N\times N}$ .

The tensor *M* can be constructed as follows:

$$M=G_{1}⊕G_{2}⊕\cdots ⊕G_{i},\;i\in 1,\ldots ,T.$$ (1)

Here, ⊕ represents the concatenation function that combines matrices into tensors along the specified dimension; in this case, dim = 0, which is particularly useful for scientific computing applications.

#### 2.2.4 Graph transformer process

The graph transformer (GT) process is inspired by the self-attention mechanism in the transformer framework (Vaswani et al., 2017). Similarly, the GT process incorporates learnable weight vectors *W* that associate and weigh the subgraphs to capture and learn more expressive and powerful node embeddings, which can be used for various graph-based learning tasks.

To associate the subgraphs and apply subgraph weighting, we introduce learnable vectors *W* represented by *W* = {*w*_1_, *w*_2_, …, *w*_*T*_}. These learnable vectors are used to perform tensor–scalar multiplication along the first dimension of *M*, which corresponds to the adjacency matrix of a subgraph *M* = {*G*_1_, *G*_2_, …, *G*_*T*_}. The multiplication of the learnable vectors *W* with the tensor *M* and subsequent dimensional accumulation results in the generation of a meta-path, denoted by *Q*:

$$Q=W\cdot M=\underset{i\in T}{\sum }\left(w_{i}\cdot G_{i}\right).$$ (2)

The elements of *w*_*i*_ are aligned with the subgraphs’ type dimension and are optimized during the training process. This approach can be considered as the core mechanism that introduces subgraph bias tailored to a specific problem. Consequently, the GT process essentially constitutes an attention-based message passing at the substructure level.

Similar to the multi-head attention mechanism in transformers, the multi-head graph transformer (MGT) process is introduced to capture and integrate diverse higher-order relationships among nodes by computing multiple meta-paths in parallel. Specifically, MGT defines a set *W* ^(*n*)^, which contains *n* weight vectors *W*, where *n* is the number of heads. During the inference process, the elements of *W* ^(*n*)^ will be calculated in parallel to generate multiple meta-paths *Q*_1_⋯*Q*_*n*_. We first define the terms of individual *Q* as *Q*_*k*_:

$$Q_{k}=W^{\left(k\right)}\cdot M=\underset{i\in T}{\sum }\left(w_{i}^{\left(k\right)}\;G_{i}\right),\;k\in 1,\ldots ,n.$$ (3)

**FIGURE 1:**
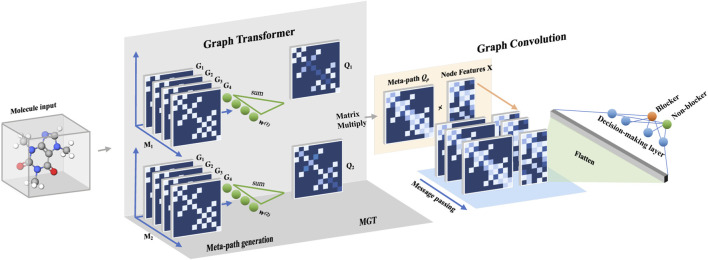
The flowchart of the GSTN model has two main stacks before the generation of the decision rule: the graph transformer (GT) and the graph convolution. In the part of GT, the first meta-graph generation step involves subgraph defining and weighing. Thereafter, a multi-head GT process is used to integrate multiple meta-paths to capture a more comprehensive relationship between the graph and the targeted property (cardiotoxicity).

At the end of the MGT process, the *Q*_*p*_ that integrates multiple meta-paths is calculated as the product of the *Q*_*k*_ terms:

$$Q_{p}=\overset{L}{\underset{k=1}{\prod }}Q_{k}=Q_{1}\cdots Q_{n},$$ (4)

where *n* also represents the *n*-hop connections that associate *Q*_1_ with *Q*_*n*_. Each *Q*_*k*_ *k* ∈ {1, …, *n*} (*n* = 2 in this study) captures different aspects of node relationships, and by combining them through multiplication, as shown in Eq. 4, the resulting *Q*_*p*_ effectively encodes essential and comprehensive information of the graph. In other words, the representation of the graph in the pipeline above contains more relevant information about the specific task, while the expressiveness of the model is enhanced by the introduction of the weighted subgraphs. The major steps of the GT process are shown in Figure 1.

#### 2.2.5 Graph convolution

After establishing the meta-path *Q*_*p*_, the graph convolution process is performed, and atomic information is passed as

$$f\left(X^{\left(l\right)},Q_{p}\right)=\sigma \left({\hat{D}}^{-\frac{1}{2}}\;\hat{Q_{p}}\;{\hat{D}}^{-\frac{1}{2}}X^{\left(l\right)}W^{\left(l\right)}\right),$$ (5)

where *f* is the function of graph convolution. $X\in R^{N\times F}$ is defined as an atomic feature matrix, where *N* is the total number of nodes in the graph and *F* is the dimensionality of the node features. *l* is the iteration counter for *f*, with *l* ∈ {1⋯*L*} representing the depth of the convolutional layers, $\hat{Q_{p}}$ is self-attended meta-path matrix, with $\hat{Q_{p}}=Q_{p}+I$ , where *I* is the identity matrix, and $\hat{D}$ is the diagonal node degree matrix of $\hat{Q_{p}}$ . The L-iteration message passing is designed with

$$X^{\left(l+1\right)}=f\left(X^{\left(l\right)},Q_{p}\right).$$ (6)

In the graph convolution process, each iteration *l* captures the local neighborhood information up to an *l*-hop distance through the meta-path *Q*_*p*_, aggregating and transforming features *X* ^(*l*)^ at each step to learn more complex and higher-order representations *X* ^(*l*+1)^ of the graph structure.

##### 2.2.5.1 Decision making

The two-dimensional matrix *X* is expanded into a one-dimensional vector *V* by flattening it according to the row-prime reshaping scheme. The resulting vector *V* with the shape of $V\in R^{1\times N\times D}$ , where *N* is the number of rows and *d* is the number of columns in matrix *X*. The flattened vector *V* is used for further computations. We designed a neural network NN, with weight matrices that are shaped as $\left\{R^{\left(N*D,h_{1}\right)},\cdots R^{\left(h_{n},2\right)}\right\}$ , *h*_*n*_ is a hidden dimension of NN, and *n* + 1 is the number of neurons layers.

### 2.3 Baseline models

To validate the improvement of the proposed model, several baseline models have been constructed. Since the GSTN model is built on the graph transformer network (GTN) model, a GTN model (Yun et al., 2019), which is actually the GSTN model without the predefined substructures, is directly compared with it. As the original graph neural network model, the molecular graph convolutional neural network (MGCNN) is used as another baseline model. In this study, an MGCNN model with two graph convolution layers and the accompanying batch normalization layers for feature extraction and fully connected layers for decision rule generation (Wu et al., 2018) was constructed. Another pervasively used molecular coding method in QSAR is the extended connectivity fingerprint (ECFP). For comparison, the ECFP (1,024 bits, diameter = 4) is input to the support vector machine (SVM) to construct a conventional QSAR model.

All of the baseline models were developed in the Python programming language by using the machine learning library Scikit-learn and the open-source cheminformatics toolkit RDKit (http://www.rdkit.org). All datasets and model implementations are available in our GitHub repository (https://github.com/huijia-wang/hERG_ChEMBL240). The preprocessed ChEMBL data and scripts for building models are also provided.

Trained with the same *hERG-DB*, a structure-based prediction with a LASSO regularized SVM model proposed by Creanza et al. (2021) is used as an additional reference outside of the QSAR modeling. The pipeline proposed by Creanza et al. differs in the input, which integrates docking scores and protein–ligand interaction fingerprints, while the SVM model was used to project the features to generate the classifying boundary.

### 2.4 Validation and evaluation

In this study, each model was trained with 10-fold cross validation and summarized with the following statistics: accuracy, precision, F1-score, recall, and area under the receiver operating characteristic curve (AUC-ROC).

$$Accuracy=\frac{TP+TN}{TP+TN+FP+FN},$$ (7)

$$Precision=\frac{TP}{TP+FP},$$ (8)

$$Recall\left(TPR\right)=\frac{TP}{TP+FN},$$ (9)

$$F1-score=\frac{2RecallPresision}{Recall+Precision}.$$ (10)

Here, *TP*, *TN*, *FP*, and *FN* are true positive, false positive, false negative, and true negative, respectively.

## 3 Results

### 3.1 Comprehensive evaluation of thresholding schemes

#### 3.1.1 Single threshold

**FIGURE 2:**
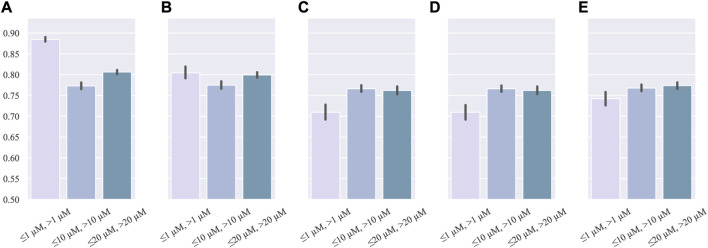
Barplot of single-threshold performance: **(A)** accuracy, **(B)** precision, **(C)**recall, **(D)** AUC, and **(E)** F1-score.

GSTN was trained with three single thresholds: *IC*_50_ = 1, 10, *and* 20 *μM*. The performance is shown in Figure 2. Even though the 1 *μM* threshold had the highest *accuracy*, its recall is lower than those of other thresholds. Additionally, in the metric of F1-score, which is suitable for assessing the performance of a model in an imbalanced dataset, *IC*_50_ = 1 *μM* has the lowest performance. In addition, all the single-threshold F1-score values are lower than 0.80. Given the discrepancy in the precision and recall, it can be inferred that the single-threshold scheme hinders the model from learning the essential features of cardiotoxicity, which is exactly the property pursued in modeling.

### 3.1.2 Dual threshold

The molecules with *IC*_50_ values in between the active and inactive thresholds can still be regarded as weak blockers and cause ambiguity in cardiotoxicity prediction, which can be generally mitigated by introducing a dual-threshold scheme. In this part, the dual thresholds will be discussed concerning 1) the effect of the different combinations of the active and inactive thresholds on removing the ambiguity of the original dataset and 2) the influence of data imbalance on the model.

**FIGURE 3:**
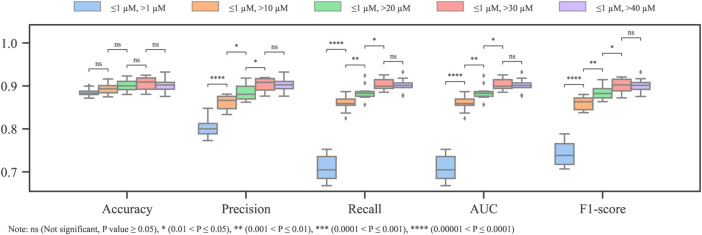
Metrics of models with different dual thresholds (imbalanced training dataset).

For the first term, after fixing the activity threshold (*IC*_50_ = 1 *μM*), different inactive thresholds (*IC*_50_ = 10, 20, 30, and 40 *μM*) were investigated. According to the Tanimoto similarity coefficient, the diversity values of all the datasets (including the original one) range between 0.768 and 0.783. Hence, it is unlikely that the removal of ambiguous molecules has a significant impact on the diversity of the dataset. As shown in Figure 3, except for the accuracy, which is substantially biased by the data imbalance, the other metrics have significant improvement after replacing the single threshold with the dual threshold. The combinations [1, 30] *μM* and [1, 40] *μM* have significantly better outcomes than the others according to the *t-test*, while no significant difference exists between them.

As the inactive threshold increases, fewer molecules can be attributed as non-blockers. Consequently, the resultant dataset in use becomes more balanced (Table 1). These results confirm that the weak blockers introduce ambiguity in the cardiotoxicity model, and the ambiguity can be greatly mitigated by weak blocker removal. In addition, the recall has the largest improvement from single threshold to dual threshold (refer to the step change of recall from single to dual threshold of [1,10] *μM* in Figure 3), it can be inferred that the ambiguity is more impeditive for the model to generate representative features for the blockers.

**FIGURE 4:**
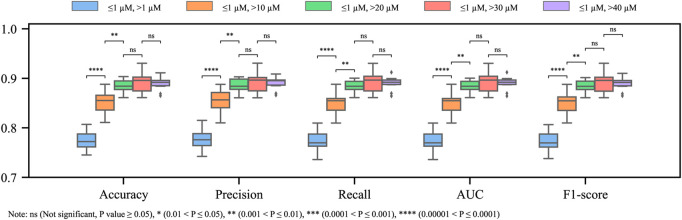
Metrics of models with different dual thresholds (balanced training dataset).

By balancing the number of blocker and non-blocker samples according to different dual thresholds, the influence of data imbalance was further investigated. In detail, for every inactive threshold, 1,295 non-blockers were selected randomly from the non-blocker class, and the number of the blockers class was constant at 1,295 (refer to Table 1 for the details). Regarding the model performance with the balanced datasets, similarly, the improvement in performance by introducing a dual threshold and enlarging the gap between the inactive and the active ones can be seen. However, the performance reaches the plateau earlier than the imbalanced one at [1, 20] *μM* (Figure 4). Combining the observations in both the balanced and imbalanced datasets, the effect of the mitigation of ambiguity from the training dataset is a positive factor and is independent of the data balancing problem.

Metric-wise cross comparisons between the imbalanced and balanced datasets are shown in Supplementary Figure S2 in the Supplementary Material, from which it can be confirmed that the adverse impact of the data imbalance is trivial. Intriguingly, the performance of the model generated with the balanced datasets is generally more stable than those generated with the imbalanced datasets in terms of the lengths of the bars and whiskers. Therefore, it is safe to conclude that the removed decoys (non-blockers) are informative for the generalizability of the model. Due to the insignificant differences between the [1, 30] *μM* and the [1, 40] *μM* in model performances, the question that which dual threshold is the most appropriate remains open at this point.

### 3.1.3 Validation with FDA-approved drugs

The FDA-approved drugs contain 45 compounds that appear in the *hERG-DB*, while the other five compounds are outside of the database. Noteworthily, as the non-active threshold increases, the number of undefined drugs, which are outside of the training set, increases (Table 2). These 50 FDA-approved drugs serve as an additional evaluation of the different thresholding schemes for the proposed method.

**TABLE 2:** Results of FDA-approved drugs on different thresholds.

No.	FDA drug name	hERG IC50 (*μ* M)	Label (≤1, >1 *μ* M threshold)	GSTN prediction	Label (≤1, >10 *μ* M threshold)	GSTN prediction	Label (≤1, >20 *μ* M threshold)	GSTN prediction	Label (≤1, >30 *μ* M threshold)	GSTN prediction	Label (≤1, >40 *μ* M threshold)	GSTN prediction
1	Desmethylastemizole	0.001	Blocker	Blocker	Blocker	Blocker	Blocker	Blocker	Blocker	Blocker	Blocker	Blocker
2	Cisapride	0.0094	Blocker	Non-blocker	Blocker	Blocker	Blocker	Blocker	Blocker	Blocker	Blocker	Blocker
3	Astemizole	0.01	Blocker	Blocker	Blocker	Blocker	Blocker	Blocker	Blocker	Blocker	Blocker	Blocker
4	Dofetilide	0.01	Blocker	Blocker	Blocker	Blocker	Blocker	Blocker	Blocker	Blocker	Blocker	Blocker
5	Sertindole	0.01	Blocker	Non-blocker	Blocker	Blocker	Blocker	Blocker	Blocker	Blocker	Blocker	Blocker
6	Haloperidol	0.0302	Blocker	Blocker	Blocker	Blocker	Blocker	Blocker	Blocker	Blocker	Blocker	Blocker
7	Droperidol	0.0324	Blocker	Blocker	Blocker	Blocker	Blocker	Blocker	Blocker	Blocker	Blocker	Blocker
8	Verapamil	0.141	Blocker	Blocker	Blocker	Blocker	Blocker	Blocker	Blocker	Blocker	Blocker	Blocker
9	Risperidone	0.151	Blocker	Non-blocker	Blocker	Blocker	Blocker	Blocker	Blocker	Blocker	Blocker	Blocker
10	***Halofantrine***	0.2	Blocker	Non-blocker	Blocker	Blocker	Blocker	Blocker	Blocker	Blocker	Blocker	Blocker
11	Terfenadine	0.2	Blocker	Blocker	Blocker	Blocker	Blocker	Blocker	Blocker	Blocker	Blocker	Blocker
12	Clozapine	0.324	Blocker	Non-blocker	Blocker	Blocker	Blocker	Blocker	Blocker	Blocker	Blocker	Blocker
13	***Quinidine***	0.324	Blocker	Non-blocker	Blocker	Non-blocker	Blocker	Non-blocker	Blocker	Blocker	Blocker	Non-blocker
14	Thioridazine	0.363	Blocker	Non-blocker	Blocker	Blocker	Blocker	Blocker	Blocker	Blocker	Blocker	Blocker
15	Bepridil	0.55	Blocker	Non-blocker	Blocker	Blocker	Blocker	Blocker	Blocker	Blocker	Blocker	Blocker
16	Vesnarinone	1.1	Non-blocker	Non-blocker	-	Non-blocker	-	Non-blocker	-	Non-blocker	-	Non-blocker
17	Azimilide	1.41	Non-blocker	Non-blocker	-	Non-blocker	-	Blocker	-	Blocker	-	Blocker
18	Mibefradil	1.45	Non-blocker	Non-blocker	-	Non-blocker	-	Blocker	-	Blocker	-	Blocker
19	Fluoxetine	1.51	Non-blocker	Non-blocker	-	Non-blocker	-	Blocker	-	Blocker	-	Non-blocker
20	Ketoconazole	1.91	Non-blocker	Non-blocker	-	Non-blocker	-	Non-blocker	-	Non-blocker	-	Blocker
21	Alosetron	3.24	Non-blocker	Non-blocker	-	Non-blocker	-	Non-blocker	-	Non-blocker	-	Non-blocker
22	Sildenafil	3.31	Non-blocker	Non-blocker	-	Non-blocker	-	Non-blocker	-	Non-blocker	-	Blocker
23	Imipramine	3.39	Non-blocker	Non-blocker	-	Blocker	-	Non-blocker	-	Non-blocker	-	Non-blocker
24	Flecainide	3.89	Non-blocker	Non-blocker	-	Non-blocker	-	Non-blocker	-	Non-blocker	-	Blocker
25	Citalopram	3.98	Non-blocker	Non-blocker	-	Blocker	-	Blocker	-	Non-blocker	-	Blocker
26	Norclozapine	4.47	Non-blocker	Non-blocker	-	Non-blocker	-	Non-blocker	-	Blocker	-	Non-blocker
27	Mosapride	4.8	Non-blocker	Non-blocker	-	Non-blocker	-	Non-blocker	-	Blocker	-	Blocker
28	Mefloquine	5.62	Non-blocker	Non-blocker	-	Non-blocker	-	Non-blocker	-	Blocker	-	Blocker
29	Cocaine	7.24	Non-blocker	Non-blocker	-	Non-blocker	-	Non-blocker	-	Blocker	-	Blocker
30	Perhexiline	7.76	Non-blocker	Non-blocker	-	Non-blocker	-	Non-blocker	-	Non-blocker	-	Non-blocker
31	Ranolazine	8.03	Non-blocker	Non-blocker	-	Non-blocker	-	Non-blocker	-	Non-blocker	-	Blocker
32	Amitriptyline	10	Non-blocker	Non-blocker	-	Non-blocker	-	Non-blocker	-	Non-blocker	-	Non-blocker
33	Nitrendipine	10	Non-blocker	Non-blocker	-	Blocker	-	Non-blocker	-	Non-blocker	-	Non-blocker
34	Carvedilol	10.5	Non-blocker	Non-blocker	Non-blocker	Non-blocker	-	Blocker	-	Blocker	-	Blocker
35	Dolasetron	12	Non-blocker	Non-blocker	Non-blocker	Non-blocker	-	Non-blocker	-	Non-blocker	-	Non-blocker
36	Diltiazem	17.4	Non-blocker	Non-blocker	Non-blocker	Non-blocker	-	Non-blocker	-	Non-blocker	-	Blocker
37	Sparfloxacin	18.2	Non-blocker	Non-blocker	Non-blocker	Non-blocker	-	Non-blocker	-	Non-blocker	-	Non-blocker
38	Pilsicainide	20.4	Non-blocker	Non-blocker	Non-blocker	Non-blocker	Non-blocker	Non-blocker	-	Non-blocker	-	Non-blocker
39	Chlorpheniramine	20.9	Non-blocker	Non-blocker	Non-blocker	Blocker	Non-blocker	Blocker	-	Non-blocker	-	Non-blocker
40	Diphenhydramine	26.9	Non-blocker	Non-blocker	Non-blocker	Non-blocker	Non-blocker	Blocker	-	Non-blocker	-	Blocker
41	Cetirizine	30.2	Non-blocker	Non-blocker	Non-blocker	Non-blocker	Non-blocker	Non-blocker	Non-blocker	Non-blocker	-	Blocker
42	Nifedipine	51	Non-blocker	Non-blocker	Non-blocker	Non-blocker	Non-blocker	Non-blocker	Non-blocker	Non-blocker	Non-blocker	Non-blocker
43	***Disopyramide***	91.2	Non-blocker	Non-blocker	Non-blocker	Non-blocker	Non-blocker	Non-blocker	Non-blocker	Non-blocker	Non-blocker	Non-blocker
44	***Epinastine***	100	Non-blocker	Non-blocker	Non-blocker	Non-blocker	Non-blocker	Non-blocker	Non-blocker	Non-blocker	Non-blocker	Blocker
45	Moxifloxacin	129	Non-blocker	Non-blocker	Non-blocker	Non-blocker	Non-blocker	Non-blocker	Non-blocker	Non-blocker	Non-blocker	Non-blocker
46	***Lidocaine***	142	Non-blocker	Non-blocker	Non-blocker	Non-blocker	Non-blocker	Non-blocker	Non-blocker	Non-blocker	Non-blocker	Non-blocker
47	***Mepivacaine***	156	Non-blocker	Non-blocker	Non-blocker	Non-blocker	Non-blocker	Non-blocker	Non-blocker	Non-blocker	Non-blocker	Non-blocker
48	***Trimethoprim***	240	Non-blocker	Non-blocker	Non-blocker	Non-blocker	Non-blocker	Non-blocker	Non-blocker	Non-blocker	Non-blocker	Blocker
49	***Nicotine***	245	Non-blocker	Non-blocker	Non-blocker	Non-blocker	Non-blocker	Non-blocker	Non-blocker	Non-blocker	Non-blocker	Non-blocker
50	***Ofloxacin***	1,410	Non-blocker	Non-blocker	Non-blocker	Non-blocker	Non-blocker	Non-blocker	Non-blocker	Non-blocker	Non-blocker	Non-blocker
Accuracy			86.0%		93.8%		89.3%		100.0%		87.5%	

Table 2 summarizes the results of GSTN models with different thresholds. Each colored block corresponds to the results with a thresholding scheme, and the first and second columns in each block show the reference with the corresponding threshold and the prediction of the GSTN model. Results in red font indicate false predictions. In addition to the five compounds which are outside of the *hERG-DB*, another four compounds are outside of the training set in the 10th-fold validation, the trained model of which is used to test the 50 drugs. These nine drugs are marked in **bold** and *italic* in Table 2. For the undefined ones due to the dual thresholds (marked as “-”), predictions of the corresponding models are supplied for further confirmation.

First, since the over-fitting has been abolished by the bias introduced by the subgraphs, data leakage does not cause a nearly perfect prediction in most cases except the [1,30] *μM* scheme. After changing the single-threshold scheme to the dual one, there is a substantial improvement. As the signal threshold is not capable of identifying some blockers (cisapride, sertindole, etc.), by using the [1,10] *μM* scheme, most of the undetected blockers can be picked up correctly. This observation suggests that by removing the weak blockers, the model becomes more sensitive to the blockers. Combined with the similar observation in the dual threshold that the ambiguity is more impeditive for the model to generate representative features for the blockers, it is safe to conclude that the dual-threshold scheme is necessary for a sensitive blocker predictor.

The model performance peaks at the [1, 30] *μM* scheme, with which all classifiable drugs can be recognized correctly. Regarding the nine external drugs, none of the thresholding schemes except for the [1, 30] *μM* can predict the blocker ***quinidine*** correctly. On the other hand, the adverse effect of the excessive removal of the non-active molecules can be seen in the results of the [1, 40] *μM* threshold. False prediction happens in both the blocker (***quinidine***) and the decoy classes (***epinastine*** and ***ofloxacin***), suggesting that the generalizability of the model is impaired by the removal. Focusing on the undefined drugs that did not appear in the training dataset, we notice that the mean *IC*_50_ values of the blocker and non-blocker prediction with the [1, 30] *μM* scheme are 4.17 and 10.14 *μM* (*t*-test *p* = 0.05), respectively. On the other side, those with the [1, 40] *μM* scheme are 9.16 and 11.04 (*t*-test *p* = 0.57), respectively. It suggests that the model with the [1, 30] *μM* scheme tends to draw a cleaner boundary for the two classes. Piecing up the results of the *hERG*-*DB* and this partially external dataset with FDA-approved drugs, a dual threshold of [1, 30] *μM* is the most appropriate for cardiotoxicity modeling.

**FIGURE 5:**
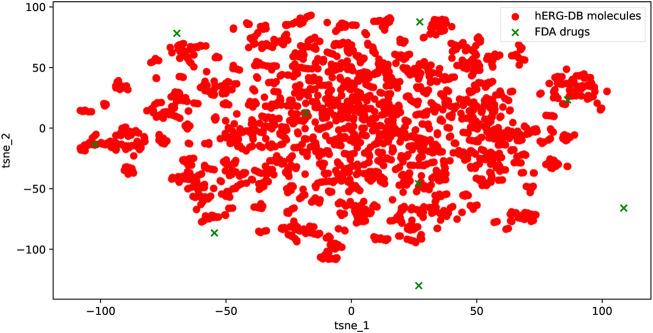
Chemical space of the training and the nine external drugs using t-SNE based on the latent features of the proposed model. The uniformity of the training and the external drugs can be seen.

For the nine external drugs in this dataset, the applicability domain (AD) is used to visualize and identify the molecules that need substantial extrapolation of the model. The latent features $\left(R^{D},D=64\right)$ generated by the Graph convolution are used as the molecular fingerprint to visualize the uniformity of the training data and the external drugs (Figure 5) by using the t-distributed stochastic neighbor embedding (t-SNE). Since the AD is used to ensure confident model prediction, chemical leverage is defined by the following equation:

$$h_{i}=x_{i}{\left(X^{T}X\right)}^{-1}x_{i}^{T}.$$ (11)

The $x_{i}\in^{D}$ represents the latent features of an external molecule, and the $X\in R^{M\times D}$ represents the fingerprints (row vector) of the training dataset (*M* the number of the molecules in the training dataset, *M* = 3347). Based on the corresponding criterion, a molecule with an *h* value that is larger than 3*D*/*M* is considered chemically different from the training dataset. Based on this idea, none of the nine drugs is identified and deemed far apart from the training dataset.

### 3.2 Predefined domain subgraphs

**FIGURE 6:**
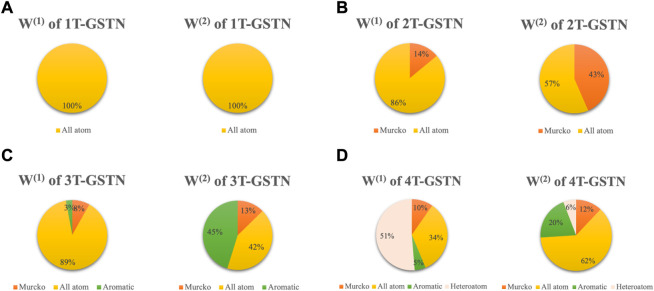
Pie plot of different combinations of subgraphs. Areas of the sectors correspond to the values of the weight of the predefined subgraphs. **(A)**: 1T-GSTN with all atoms predefined domain subgraph; **(B)**: 2T-GSTN with all-atoms and Murcko subgraphs; **(C)**: 3T-GSTN with all-atoms, Murcko, and aromatic ring subgraphs; and **(D)**: 4T-GSTN with all predefined subgraphs.

**FIGURE 7:**
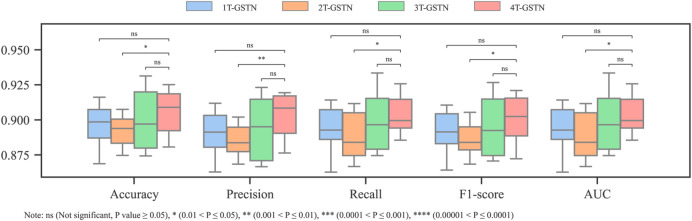
Performance of different combinations of subgraphs.

The effect of predefined subgraphs is investigated by looking into the learnable weights *W* ^(1)^ and *W* ^(2)^. Figure 6 shows a direct comparison of different predefined subgraph combinations; Figure 7 shows the performance of the models with a different subgraph bias. 2T-GSTN is the model with all-atoms and Murcko subgraphs; 3T-GSTN is with all-atoms, Murcko, and aromatic ring subgraphs; and 4T-GSTN is with all predefined subgraphs. Although no significant improvement, a slight improvement over the graph transformer (1T-GSTN) in the accuracy and F1-score can be seen in the 4T-GSTN model. In Figure 6, the learnable weights of different subgraphs combinations are averaged over the 10-fold cross validation, which suggests the necessity of importing the predefined substructures.

Noteworthily, 2T-GSTN shows an inferior performance when compared to others. It may be caused by the high similarity between the scaffold and the molecule. On the other hand, the necessity of the ring and heteroatom can be seen in Figure 6. The importance of the heteroatom is consistent with the findings of previous studies (Creanza et al. (2021); Moorthy et al. (2021); Kim and Nam (2020)).

### 3.3 Performance comparison

**FIGURE 8:**
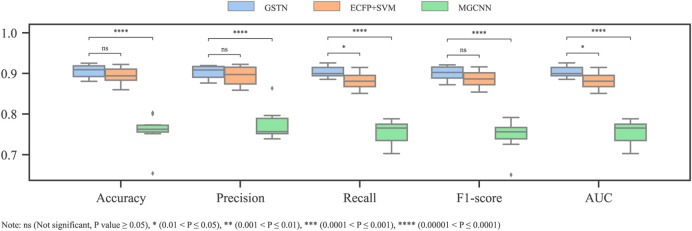
Comparison performance with baseline models.

By applying the 10-fold cross validation to both the GSTN model and the baseline models, a comparison with the baseline models is summarized in Figure 8. Comparing the GSTN model with the one based on MGCNN, the manipulation of the meta-path show its superiority in representing the molecular graph as a heterogeneous graph. The superiority of the GSTN model can be also seen in the time duration of training. Because of the parallel processing in the GT process, the training time is 40 s (100 epochs) in our model, while that in the MGCNN model is 640 s using a MacBook Pro with a 12-core CPU and an 18-core GPU.

With respect to the other baseline model, the SVM model input with the ECFP, its inferior performance compared to that of the GTSN model suggests that the global context of the substructures, which is neglected by the ECFP, is beneficial for the cardiotoxicity modeling.

Albeit with the same training dataset, the input of the model proposed by Creanza et al. is more microscopic and deterministic. Docking scores of the *hERG* central cavity and the protein–ligand interaction fingerprint are integrated as the input. Although the validation scheme is not clear in Creanza’s study, given that similar dual-threshold schemes are used and the 10-fold cross validation in our study is a standard way for model evaluation, it is plausible to say that the GTSN model has a better performance in terms of accuracy and AUC values.

## 4 Discussion

### 4.1 On the activity cliff corner

The efficacy of the GSTN model in harnessing the activity cliff problem, which is a nuisance in QSAR modeling, was investigated in this section by comparing the proposed model with the baseline models. The comparison centered on the common/unique Murcko scaffold is conducted. The Murcko scaffold represents the union of the rings and liners in a molecule (Bemis and Murcko, 1996). Although it may be insufficient in highlighting all the important features, the Murcko scaffold is generally used to find the structure similarity of molecules. Therefore, the investigation is discussed in the following three different directions.

#### 4.1.1 Common Murcko scaffold with different properties

Common Murcko scaffold with different properties means that, albeit with the same Murcko scaffold, molecules can be either blockers or non-blockers. We summarized six major groups in Table 3, from which it can be confirmed that the GTSN model handles the activity cliff better than the baseline models. For example, albeit with balanced numbers of blockers and non-blockers in group 1, the MGCNN and ECFP with SVM models fail at recognizing the blocker in the test dataset. Intriguingly, although being trained with the non-blockers only, the GSTN model is able to pick up the subtle difference between blockers and non-blocker, for which reason the GSTN model can recognize the blocker in group 2 while the other two models fail to do so.

**TABLE 3:** Common Murcko scaffold molecules with different properties.

Group	No.	Canonical_smiles	Murcko	Class	GSTN	MGCNN	ECFP + SVM	Test/training
	1	OC(=O)c1ccc(cc1)C2 = CC3(CCNCC3)Oc4ccccc24	C1 = C(c2ccccc2)c2ccccc2OC12CCNCC2	Non-blocker				Training
	2	C1CC2(CCN1)Oc3ccccc3C(=C2)c4ccccc4	C1 = C(c2ccccc2)c2ccccc2OC12CCNCC2	Blocker				Training
	3	Oc1ccc(cc1)C2 = CC3(CCNCC3)Oc4ccccc24	C1 = C(c2ccccc2)c2ccccc2OC12CCNCC2	Blocker				Training
	4	CCN(CC)C(=O)c1ccc(C2 = CC3(CCNCC3)Oc4ccccc24)c(O)c1	C1 = C(c2ccccc2)c2ccccc2OC12CCNCC2	Non-blocker				Training
	5	CCN(CC)C(=O)c1ccc(cc1)C2 = CC3(CCN(CC3)C(=O)C)Oc4ccccc24	C1 = C(c2ccccc2)c2ccccc2OC12CCNCC2	Non-blocker				Training
	6	Cc1ccc(cc1)C2 = CC3(CCNCC3)Oc4ccccc24	C1 = C(c2ccccc2)c2ccccc2OC12CCNCC2	Blocker				Training
	7	CCN(CC)C(=O)c1ccc(cc1)C2 = CC3(CCNCC3)Oc4cc(O)ccc24	C1 = C(c2ccccc2)c2ccccc2OC12CCNCC2	Non-blocker				Training
	8	COc1ccc(cc1)C2 = CC3(CCNCC3)Oc4ccccc24	C1 = C(c2ccccc2)c2ccccc2OC12CCNCC2	Blocker				Training
	9	CCN(CC)C(=O)c1ccc(cc1)C2 = CC3(CCN(C)CC3)Oc4ccccc24	C1 = C(c2ccccc2)c2ccccc2OC12CCNCC2	Blocker				Training
	10	CNC(=O)c1ccc(cc1)C2 = CC3(CCNCC3)Oc4ccccc24	C1 = C(c2ccccc2)c2ccccc2OC12CCNCC2	Non-blocker				Training
	11	CCN(CC)C(=O)c1ccc(C2 = CC3(CCNCC3)Oc4ccccc24)c(F)c1	C1 = C(c2ccccc2)c2ccccc2OC12CCNCC2	Blocker				Training
	12	CCN(CC)C(=O)c1ccc(cc1)C2 = CC3(CCNCC3)Oc4cccc(O)c24	C1 = C(c2ccccc2)c2ccccc2OC12CCNCC2	Non-blocker				Training
	13	FC(F)(F)c1ccc(cc1)C2 = CC3(CCNCC3)Oc4ccccc24	C1 = C(c2ccccc2)c2ccccc2OC12CCNCC2	Blocker				Training
	14	COC(=O)c1ccc(cc1)C2 = CC3(CCNCC3)Oc4ccccc24	C1 = C(c2ccccc2)c2ccccc2OC12CCNCC2	Blocker				Training
	15	N#Cc1ccc(cc1)C2 = CC3(CCNCC3)Oc4ccccc24	C1 = C(c2ccccc2)c2ccccc2OC12CCNCC2	Blocker				Training
1	16	CCN(CC)C(=O)c1cccc(c1)C2 = CC3(CCNCC3)Oc4ccccc24	C1 = C(c2ccccc2)c2ccccc2OC12CCNCC2	Blocker	Pass	Fail	Fail	Test
**Group**	**No.**	**Canonical_smiles**	**Murcko**	**Class**	**GSTN**	**MGCNN**	**ECFP + SVM**	**Test/training**
	1	CC(C)S(=O)(=O)N[C@H]1CN(C)C[C@@H]1c2ccc(cc2)c3cccc(c3)S(=O)(=O)C	c1ccc(-c2ccc(C3CCNC3)cc2)cc1	Non-blocker				Training
	2	CC(C)S(=O)(=O)N[C@H]1CN(C)C[C@@H]1c2ccc(cc2)c3cccc(NS(=O)(=O)C)c3	c1ccc(-c2ccc(C3CCNC3)cc2)cc1	Non-blocker				Training
2	3	CCN1C[C@H](NS(=O)(=O)C(C)C)[C@H](C1)c2ccc(cc2)c3ccc(cc3)C#N	c1ccc(-c2ccc(C3CCNC3)cc2)cc1	Blocker	Pass	Fail	Fail	Test
**Group**	**No.**	**Canonical_smiles**	**Murcko**	**Class**	**GSTN**	**MGCNN**	**ECFP + SVM**	**Test/training**
	1	CC#CC(CC(=O)O)c1ccc(Oc2ccc(cc2OC(F)F)C(F)(F)F)cc1	c1ccc(Oc2ccccc2)cc1	Non-blocker				Training
	2	CS(=O)(=O)Nc1ccc(cc1Oc2ccccc2)[N+](=O)[O-]	c1ccc(Oc2ccccc2)cc1	Non-blocker				Training
	3	CC(C(=O)O)c1cccc(Oc2ccccc2)c1	c1ccc(Oc2ccccc2)cc1	Non-blocker				Training
	4	CNCc1ccc(OC)cc1Oc2ccc(Cl)c(Cl)c2	c1ccc(Oc2ccccc2)cc1	Blocker				Training
	5	CNC(C)c1ccc(OC)cc1Oc2ccc(Cl)c(Cl)c2	c1ccc(Oc2ccccc2)cc1	Blocker				Training
	6	CC(N(C)C)c1ccccc1Oc2ccc(Cl)c(Cl)c2	c1ccc(Oc2ccccc2)cc1	Blocker				Training
	7	CNCc1ccc(Br)cc1Oc2ccc(Cl)c(Cl)c2	c1ccc(Oc2ccccc2)cc1	Blocker				Training
	8	COc1ccc(CN(C)C)c(Oc2ccc(Cl)c(Cl)c2)c1	c1ccc(Oc2ccccc2)cc1	Blocker				Training
	9	CNCc1ccc(Cl)cc1Oc2ccc(Cl)cc2	c1ccc(Oc2ccccc2)cc1	Blocker	Pass	Fail	Pass	Test
3	10	CNCc1ccc(Cl)cc1Oc2ccc(F)c(c2)C(F)(F)F	c1ccc(Oc2ccccc2)cc1	Blocker	Pass	Pass	Pass	Test
**Group**	**No.**	**Canonical_smiles**	**Murcko**	**Class**	**GSTN**	**MGCNN**	**ECFP + SVM**	**Test/training**
	1	CC(C)S(=O)(=O)N[C@H]1CN(C)C[C@@H]1c2ccc(cc2)c3ccc(F)nc3	c1cncc(-c2ccc(C3CCNC3)cc2)c1	Blocker				Training
	2	CC(C)S(=O)(=O)N[C@H]1CN(C)C[C@@H]1c2ccc(cc2)c3cccnc3	c1cncc(-c2ccc(C3CCNC3)cc2)c1	Non-blocker	Pass	Fail	Pass	Test
4	3	CC(C)S(=O)(=O)N[C@@H]1COC[C@@H]1c2ccc(cc2)c3cncc(F)c3	c1cncc(-c2ccc(C3CCOC3)cc2)c1	Non-blocker				Training
**Group**	**No.**	**Canonical_smiles**	**Murcko**	**Class**	**GSTN**	**MGCNN**	**ECFP + SVM**	**Test/training**
	1	CN(C)C(=O)c1ccc(c(F)c1)c2ccc3C(=O)N(CCN4CCCC4)CCc3c2	O=C1c2ccc(-c3ccccc3)cc2CCN1CCN1CCCC1	Non-blocker				Training
	2	C[C@@H]1CCCN1CCN2CCc3cc(ccc3C2 = O)c4ccc(cc4)C#N	O=C1c2ccc(-c3ccccc3)cc2CCN1CCN1CCCC1	Blocker				Training
5	3	C[C@@H]1CCCN1CCN2CCc3cc(ccc3C2 = O)c4ccc(F)cc4	O=C1c2ccc(-c3ccccc3)cc2CCN1CCN1CCCC1	Blocker	Pass	Fail	Pass	Test
**Group**	**No.**	**Canonical_smiles**	**Murcko**	**Class**	**GSTN**	**MGCNN**	**ECFP + SVM**	**Test/training**
	1	FC(F)(F)c1ccc(cc1)S(=O)(=O)Nc2ccc(cc2)[C@]34CNC[C@H]3C4	O=S(=O)(Nc1ccc(C23CNCC2C3)cc1)c1ccccc1	Blocker				Training
	2	CN(c1ccc(cc1)[C@]23CNC[C@H]2C3)S(=O)(=O)c4ccccc4	O=S(=O)(Nc1ccc(C23CNCC2C3)cc1)c1ccccc1	Non-blocker				Training
	3	CC(C)c1ccc(cc1)S(=O)(=O)Nc2ccc(cc2)[C@]34CNC[C@H]3C4	O=S(=O)(Nc1ccc(C23CNCC2C3)cc1)c1ccccc1	Blocker				Training
6	4	CC(C)c1ccc(cc1)S(=O)(=O)Nc2ccc(cc2)[C@@]34C[C@@H]3CN(CC = C)C4	O=S(=O)(Nc1ccc(C23CNCC2C3)cc1)c1ccccc1	Blocker	Pass	Pass	Fail	Test

Given that ECFP with the SVM model showed no high sensitivity in predicting the molecules with the same Murcko but different properties, it is safe to conclude that the GTSN model proposed in this work is more capable of picking up the decisive structural features for the cardiotoxicity problem.

#### 4.1.2 Common Murcko scaffold with the same property

Contrarily, a model putting too much weight on the side chains may lead to an over-fitting problem. In this regard, the molecules with the same scaffold and the same cardiotoxicity properties are extracted, as shown in Table 4. For the eight major groups shown in Table 4, the GSTN recognizes the properties of the test molecules in each group.

**TABLE 4:** Common Murcko scaffold molecule with the same properties.

Group	No.	Canonical_smiles	Murcko	Class	GSTN	MGCNN	ECFP + SVM	Test/training
	1	CCOC(=O)N1CCN(Cc2oc(nn2)c3cc(Cl)ccc3F)CC1	c1ccc(-c2nnc(CN3CCNCC3)o2)cc1	Non-blocker				Training
1	2	CCOC(=O)N1CCN(Cc2oc(nn2)c3cccc(Cl)c3)CC1	c1ccc(-c2nnc(CN3CCNCC3)o2)cc1	Non-blocker	Pass	Fail	Fail	Test
**Group**	**No.**	**Canonical_smiles**	**Murcko**	**Class**	**GSTN**	**MGCNN**	**ECFP + SVM**	**Test/training**
	1	CN(C)c1cn(c2ccc(F)cc2)c3ccc(Cl)cc13	c1ccc(-n2ccc3ccccc32)cc1	Blocker				Training
2	2	CN(C)Cc1cn(c2ccc(F)cc2)c3ccc(Cl)cc13	c1ccc(-n2ccc3ccccc32)cc1	Blocker	Pass	Fail	Pass	Test
**Group**	**No.**	**Canonical_smiles**	**Murcko**	**Class**	**GSTN**	**MGCNN**	**ECFP + SVM**	**Test/training**
	1	CSc1ccc2Sc3ccccc3N(CCC4CCCCN4C)c2c1	c1ccc2c(c1)Sc1ccccc1N2CCC1CCCCN1	Blocker				Training
3	2	CN1CCCCC1CCN2c3ccccc3Sc4ccc(cc24)[S+](C)[O-]	c1ccc2c(c1)Sc1ccccc1N2CCC1CCCCN1	Blocker	Pass	Fail	Fail	Test
**Group**	**No.**	**Canonical_smiles**	**Murcko**	**Class**	**GSTN**	**MGCNN**	**ECFP + SVM**	**Test/training**
	1	COC(=O)N1CCN(CC1)c2ccc(Nc3ncc(Cl)c(n3)c4cnc5ccccn45)c(OC)c2	c1ccn2c(-c3ccnc(Nc4ccc(N5CCNCC5)cc4)n3)cnc2c1	Non-blocker				Training
	2	COc1cc(ccc1Nc2ncc(Cl)c(n2)c3cnc4ccccn34)N5CCN(CC5)C(=O)N	c1ccn2c(-c3ccnc(Nc4ccc(N5CCNCC5)cc4)n3)cnc2c1	Non-blocker				Training
4	3	COc1cc(ccc1Nc2ncc(Cl)c(n2)c3cnc4ccccn34)N5CCN(CC5)S(=O)(=O)C	c1ccn2c(-c3ccnc(Nc4ccc(N5CCNCC5)cc4)n3)cnc2c1	Non-blocker	Pass	Fail	Pass	Test
**Group**	**No.**	**Canonical_smiles**	**Murcko**	**Class**	**GSTN**	**MGCNN**	**ECFP + SVM**	**Test/training**
	1	FC(F)(F)c1ccccc1C(=O)N2CCC(Cc3ccccc3)CC2	O=C(c1ccccc1)N1CCC(Cc2ccccc2)CC1	Non-blocker				Training
	2	Cc1ccccc1C(=O)N2CCC(Cc3ccccc3)CC2	O=C(c1ccccc1)N1CCC(Cc2ccccc2)CC1	Non-blocker				Training
	3	Clc1ccccc1C(=O)N2CCC(Cc3ccccc3)CC2	O=C(c1ccccc1)N1CCC(Cc2ccccc2)CC1	Non-blocker				Training
	4	Fc1ccccc1C(=O)N2CCC(Cc3ccccc3)CC2	O=C(c1ccccc1)N1CCC(Cc2ccccc2)CC1	Non-blocker				Training
5	5	COc1ccccc1C(=O)N2CCC(Cc3ccccc3)CC2	O=C(c1ccccc1)N1CCC(Cc2ccccc2)CC1	Non-blocker	Pass	Fail	Pass	Test
**Group**	**No.**	**Canonical_smiles**	**Murcko**	**Class**	**GSTN**	**MGCNN**	**ECFP + SVM**	**Test/training**
	1	CC1(C)Cc2cccc(CN3CCC4(CC3)CCN(CC4)C(=O)c5ccc(N)cn5)c2O1	O=C(c1ccccn1)N1CCC2(CCN(Cc3cccc4c3OCC4)CC2)CC1	Non-blocker	Pass	Fail	Pass	Training
6	2	CC1(C)Oc2c(CN3CCC4(CC3)CCN(CC4)C(=O)c5ccc(N)cn5)cccc2C1(F)F	O=C(c1ccccn1)N1CCC2(CCN(Cc3cccc4c3OCC4)CC2)CC1	Non-blocker	Pass	Fail	Pass	Test
**Group**	**No.**	**Canonical_smiles**	**Murcko**	**Class**	**GSTN**	**MGCNN**	**ECFP + SVM**	**Test/training**
	1	COc1ccc2C = CC(=O)N(CCN3CCC(CC3)NCc4cc5OCCOc5cn4)c2c1	O = c1ccc2ccccc2n1CCN1CCC(NCc2cc3c(cn2)OCCO3)CC1	Non-blocker				Training
	2	O=C1C = Cc2ccc(cc2N1CCN3CCC(CC3)NCc4cc5OCCOc5cn4)C#N	O = c1ccc2ccccc2n1CCN1CCC(NCc2cc3c(cn2)OCCO3)CC1	Non-blocker				Training
7	3	F[C@H]1CN(CCN2C(=O)C=Cc3ccc(cc23)C#N)CC[C@@H]1NCc4cc5OCCOc5cn4	O = c1ccc2ccccc2n1CCN1CCC(NCc2cc3c(cn2)OCCO3)CC1	Non-blocker	Pass	Fail	Pass	Test
**Group**	**No.**	**Canonical_smiles**	**Murcko**	**Class**	**GSTN**	**MGCNN**	**ECFP + SVM**	**Test/training**
	1	COC(=O)c1ccc2ncc(F)c(CCC34CCC(CC3)(CO4)NCc5ccc6OCC(=O)Nc6n5)c2n1	O=C1COc2ccc(CNC34CCC(CCc5ccnc6cccnc56)(CC3)OC4)nc2N1	Blocker				Training
	2	FC(F)Oc1ccc2ncc(F)c(CCC34CCC(CC3)(CO4)NCc5ccc6OCC(=O)Nc6n5)c2n1	O=C1COc2ccc(CNC34CCC(CCc5ccnc6cccnc56)(CC3)OC4)nc2N1	Blocker				Training
	3	COc1cc2ncc(F)c(CCC34CCC(CC3)(CO4)NCc5ccc6OCC(=O)Nc6n5)c2nc1OC	O=C1COc2ccc(CNC34CCC(CCc5ccnc6cccnc56)(CC3)OC4)nc2N1	Blocker				Training
	4	COc1ccc2nc(OC)cc(CCC34CCC(CC3)(CO4)NCc5ccc6OCC(=O)Nc6n5)c2n1	O=C1COc2ccc(CNC34CCC(CCc5ccnc6cccnc56)(CC3)OC4)nc2N1	Blocker				Training
	5	COc1ccc2ncc(c(CCC34CCC(CC3)(CO4)NCc5ccc6OCC(=O)Nc6n5)c2n1)C(F)(F)F	O=C1COc2ccc(CNC34CCC(CCc5ccnc6cccnc56)(CC3)OC4)nc2N1	Blocker				Training
	6	Fc1cnc2ccc(nc2c1CCC34CCC(CC3)(CO4)NCc5ccc6OCC(=O)Nc6n5)C#N	O=C1COc2ccc(CNC34CCC(CCc5ccnc6cccnc56)(CC3)OC4)nc2N1	Blocker				Training
	7	COc1ccc2ncc(F)c(CCC34CCC(CC3)(CO4)NCc5ccc6OCC(=O)N(C)c6n5)c2n1	O=C1COc2ccc(CNC34CCC(CCc5ccnc6cccnc56)(CC3)OC4)nc2N1	Blocker	Pass	Pass	Pass	Test
	8	Cc1cc(nc2c(CCC34CCC(CC3)(CO4)NCc5ccc6OCC(=O)Nc6n5)ccnc12)S(=O)(=O)C	O=C1COc2ccc(CNC34CCC(CCc5ccnc6cccnc56)(CC3)OC4)nc2N1	Blocker	Pass	Pass	Pass	Test
8	9	CCc1c(C)c2OCC(=O)Nc2nc1CNC34CCC(CCc5c(F)cnc6ccc(OC)nc56)(CC3)OC4	O=C1COc2ccc(CNC34CCC(CCc5ccnc6cccnc56)(CC3)OC4)nc2N1	Blocker	Pass	Fail	Pass	Test

#### 4.1.3 Unique Murcko scaffold

It is not unusual that some bioactivity can be largely decided by the chemical scaffold. There is also a portion of molecules in our dataset, whose cardiotoxicity properties can be decided by their scaffolds. Hence, the question “does the local structure-based algorithms (both ECFP and MGCNN) grasp the scaffold information concretely or not?” has a direct impact on their performance in this regard. We, therefore, further investigate the models’ performances on the unique Murcko scaffold, which means that no molecule with the same scaffold as the one of interest is used in the training dataset.

From Table 5, it can be confirmed that the GSTN model outperforms the two baseline models by a large margin. Of note, the GSTN model also uses a GCN layer to extract and summarize the features of the local structures of the final meta-graph. The superior performance may come from the weighted attention manipulation introduced by the predefined substructures.

**TABLE 5:** Unique Murcko scaffold.

No.	Canonical_smiles	Murcko	Class	GSTN	MGCNN	ECFP + SVM	Test
1	C[C@@H]1CCCN1CCc2ccc3nc(ccc3c2)c4ccn[nH]4	c1cc(-c2ccc3cc(CCN4CCCC4)ccc3n2)[nH]n1	Non-blocker	Pass	Pass		Test
2	CN(C)CCCCc1ccc(cc1)C2(C)COC2	c1ccc(C2COC2)cc1	Non-blocker	Pass	Pass	Fail	Test
3	CCOc1nc2cccc(C(=O)O)c2n1Cc3ccc(cc3)c4ccccc4c5nnn[nH]5	c1ccc(-c2nnn[nH]2)c(-c2ccc(Cn3cnc4ccccc43)cc2)c1	Non-blocker	Pass		Pass	Test
4	Cl.Nc1ncc(cc1c2oc3ccccc3n2)c4cnn(c4)C5CCNCC5	c1ccc2oc(-c3cncc(-c4cnn(C5CCNCC5)c4)c3)nc2c1	Blocker	Pass	Pass	Fail	Test
5	C(COc1ccc(CCCN2CCN(CC2)c3cccc4cccnc34)cc1)CN5CCCCCC5	c1cnc2c(N3CCN(CCCc4ccc(OCCCN5CCCCCC5)cc4)CC3)cccc2c1	Blocker	Pass	Pass	Fail	Test
6	NC(=O)c1cnc2[nH]ccc2c1NC3CCN(CC3)c4ccc(nn4)C#N	c1cnnc(N2CCC(Nc3ccnc4[nH]ccc34)CC2)c1	Non-blocker	Pass	Fail	Pass	Test
7	CC(C)(C)c1ccc(NC(=O)N2CCN(CC2)c3ncccc3Cl)cc1	O=C(Nc1ccccc1)N1CCN(c2ccccn2)CC1	Blocker	Pass	Fail	Fail	Test
8	Nc1nccn2c(nc(c3ccc(cc3F)C(=O)Nc4cc(ccn4)C(F)(F)F)c12)[C@@H]5CCCN(C5)C6CCOCC6	O=C(Nc1ccccn1)c1ccc(-c2nc(C3CCCN(C4CCOCC4)C3)n3ccncc23)cc1	Blocker	Pass	Fail	Fail	Test
9	CC(C)C(=O)NCc1cnc(C(F)F)c(c1)C(=O)Nc2nc(C)c([nH]2)c3ccc(cc3)C(F)(F)F	O=C(Nc1ncc(-c2ccccc2)[nH]1)c1cccnc1	Blocker	Pass	Fail	Fail	Test
10	O=C1Nc2ccccc2N1C3CCN(CC3)C4CCCCC4	O = c1[nH]c2ccccc2n1C1CCN(C2CCCCC2)CC1	Non-blocker	Pass	Fail	Pass	Test
11	Cc1c2CCCN3CCCC[C@@H]3CNc4cc(ccc4C(=O)N)n2c5CC(C)(C)CC(=O)c15	O=C1CCCc2c1cc1n2-c2cccc(c2)NCC2CCCCN2CCC1	Blocker	Pass	Fail	Fail	Test
12	CN(C)CCCn1cc(C2 = C(C(=O)NC2 = O)c3c[nH]c4ccccc34)c5ccccc15	O=C1NC(=O)C(c2c[nH]c3ccccc23) = C1c1c[nH]c2ccccc12	Blocker	Pass	Fail	Pass	Test
13	COc1ccc2[C@H](OC(=O)c2c1OC)[C@@H]3N(C)CCc4cc5OCOc5c(OC)c34	O=C1OC(C2NCCc3cc4c(cc32)OCO4)c2ccccc21	Non-blocker	Pass	Fail	Pass	Test
14	Cc1c2COC(=O)c2ccc1[C@@H](O)CN3CCC4(CC3)CCN(CC4)c5ccc(cn5)C#N	O=C1OCc2cc(CCN3CCC4(CC3)CCN(c3ccccn3)CC4)ccc21	Blocker	Pass	Pass	Fail	Test

### 4.2 Model interpretability

**FIGURE 9:**
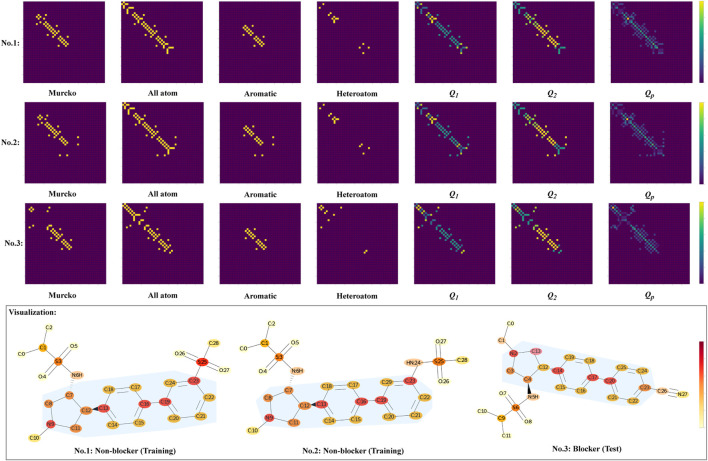
Visualization of the three molecules with the same scaffold but different cardiotoxicity properties. The meanings of *Q*_1_, *Q*_2_, and *Q*_*p*_ can be found in the description of the GSTN model. The area with blue shading represents the Murcko scaffold.

By visualization of the subgraphs and the resultant *Q*_*p*_, the GSTN model provides additional information for the compound design. Alongside the four subgraphs, the resultant *Q*_1_, *Q*_2_, and the final *Q*_*p*_ were extracted. As a newly generated weighted graph for the subsequent graph convolution, the *Q*_*p*_ is used for model interpretation. Given the differences in the cardiotoxicity property, the molecules of group 2 in Table 3 are used as an example, and the no. 1, 2, and 3 molecules in Figure 9 correspond to the molecules with the same group number, respectively.

In each predefined subgraph, different bonds are unified (see the grids for each substructure in Figure 9), and the attention of substructure-level heterogeneity is introduced by the multiplication with weights, as can be seen in the *Q*_1_, *Q*_2_, and the resultant *Q*_*p*_. Moreover, the no. 3 molecule (blocker) clearly shows a distinct *Q*_*p*_ from the other two non-blockers. Specifically, albeit with the same scaffold, the resultant connectivity shows different patterns in attention (especially in the connection marked with yellow color).

The grid diagram in Figure 9 provides a more intuitive view of the model, where the scaffold is indicated by the light blue shadow. Generally, the three molecules have highly similar structures. For example, the sulfonamide functional group is connected to the 7th position of the carbon atoms in the no. 1 and no. 2 molecules, as well as to the 4th carbon atom of the no. 3 molecule. For all this, the model is able to recognize the subtle difference, which is the difference in the substituents of the 23rd carbon atoms, by devoting sufficient attention to that carbon. In particular, the no. 3 molecule features an alkynyl group located in the paraposition of the benzene ring, while the other two are in the metaposition.

### 4.3 On the thresholding scheme

The necessity of the dual threshold is reconfirmed in our study. Although a portion of the samples will become undefined, the performance of the prediction is improved significantly. Specifically, the sensitivity to the blocker (recall) is lifted from 0.71 to 0.86 by introducing the [1, 10] *μM* dual-thresholding scheme and peaks at 0.90 with [1, 30] *μM* threshold. This improvement is desired in the pre-screen step.

Another concern about the dataset is the data balance. Generally, data balancing is beneficial to minor classes (the class with a smaller number of samples) in deep learning. However, the results of this study show that adverse influence from the mild data imbalance is trivial, and excessive removal of the samples in the decoy classes will impair the generalizability of the model. Based on the aforestated discussion, the imbalanced dataset with the [1, 30] *μM* is recommended. This thresholding scheme is consistent with the previous study. In Carvalho’s study, it is suggested for a safety margin, the *IC*_50_ of the non-toxic threshold should be at least 30-fold of the toxic one (Carvalho et al., 2013). Given that the 1 *μM* is usually used as the threshold of hERG blockers, 30 *μM* is appropriate to be set as the safety threshold (Li et al., 2017).

The introduction of the predefined substructures shows its advantage over the original graph transformer. Without significant improvement, the GSTN model returns the results with slightly higher median values and smaller variations for all metrics. Given that the bias of the substructures is introduced by the learnable weights, the model is with good scalability, based on which new biochemical insight can be integrated into the model easily.

### 4.4 On the models

The insufficiency of the ECFP-based model in coping with the unique Murcko scaffold (Table 5) can be understood according to the theory of ECFP. By embedding the radius-wise substructure into fixed-length bit strings, the order of the strings is rearranged according to the order of the original identifiers for substructures (Morgan, 1965), for which the long scaffold may not be preserved appropriately, and the connection information between substructures cannot be considered properly in its one-hot vector (Rogers and Hahn, 2010).

The inferior performance of the MGCNN model suggests the necessity of heterogeneity at a certain level. Noteworthily, the heterogeneity is not expressed in the level of chemical bond but in the level of predefined substructure, as explained above, which may be more effective in the biochemical domain.

A computation model that is based on docking scores is regarded as a highly efficient way to assess the hERG–drug interaction (Koulgi et al., 2021). In the reference study, the docking score is further integrated with the protein–ligand interaction fingerprints to characterize the behavior of small molecules in the binding site of target proteins and to elucidate fundamental biochemical processes. However, at the current stage, given the low resolution (3.7 Å) and the absence of a ligand, it is difficult to construct an atomic-level model of the protein that reflects the site conformational rearrangement. In addition, the best structure-based model ends with a 0.71 accuracy on 5VA1-IFD-4 conformations (with [1, 30] *μM* threshold).

In our study, there is a portion of undefined weak blockers in the dual-threshold setting. Further investigation around the weak blockers using another scheme, such as the regression, can be very interesting.

## 5 Conclusion

In this study, a new graph neural model was proposed by introducing the substructure-aware bias to solve the cardiotoxicity prediction problem. Based on domain knowledge, four types of substructures were used to extract the key feature of cardiotoxic molecules and to strengthen the robustness toward the active cliff problem. Combined with the ≤1μM,>30μM dual-thresholding scheme, which is validated by a comprehensive comparison with the *hERG*-*DB* and the partially external FDA-approved drugs dataset, the best model attains performance with 90.5% accuracy, 90.4% precision, 90.4% recall, 90.5% F1-score, and 90.4% AUC. Based on comparisons with the baseline models, the improved pipeline (GSTN model and thresholding scheme) has been validated in terms of the activity cliff problem as well. As a final benefit, the proposed pipeline enables medical researchers to visualize key aspects of cardiotoxicity molecules in order to better understand them.

## Data Availability

Publicly available datasets were analyzed in this study. These data can be found at: https://pubs.acs.org/doi/10.1021/acs.jcim.1c00744?goto=supporting-info.
