# Supplementary material for: On QSAR-based cardiotoxicity modeling with the expressiveness-enhanced graph learning model and dual-threshold scheme
Source: Front Physiol. 2023 May 9;14:1156286. doi: 10.3389/fphys.2023.1156286 (PMC10203956; doi:10.3389/fphys.2023.1156286)
Supplement: Supplementary file 1 [file Presentation1.pdf]

Supplementary Material

1 HERG-DB

ChEMBL is a bioactivity database maintained by the European Bioinformatics Institute and is frequently used in various cheminformatics research as the *de facto* standard database Sato et al. (2018). Therefore, the hERG dataset used in this study from Creanza's Creanza et al. (2021) and the ChEMBL bioactivity database Gaulton et al. (2012). It provides more than 20,000 molecules related to the hERG protein channel ("target_ID" = "ChEMBL240"). We selected data from human or biologically similar assays (HEK, CHO) annotated with IC_{50} as a standard method to assess hERG blocker ("target_organism" ="Homo sapiens"). It is acceptable for a classification task to tolerate noise caused by hERG IC_{50} variability Wang et al. (2016). Data marked as direct binding ("assay_type" = "B"), which means binding to ion channel protein method. It is mainly based on the manual patch clamp method, a gold standard for ion channel functional evaluation. Remove warnings information in the "data_validity_comment", and "No Data" in "standard_value" and duplicates. The distribution of pIC50 (-log IC50) of the HERG-DB is shown in Figure S1

REFERENCES

Creanza, T. M., Delre, P., Ancona, N., Lentini, G., Saviano, M., and Mangiatordi, G. F. (2021). Structure-based prediction of herg-related cardiotoxicity: A benchmark study. *Journal of Chemical Information and Modeling* 61, 4758–4770

Gaulton, A., Bellis, L. J., Bento, A. P., Chambers, J., Davies, M., Hersey, A., et al. (2012). Chembl: a large-scale bioactivity database for drug discovery. *Nucleic acids research* 40, D1100–D1107

Sato, T., Yuki, H., Ogura, K., and Honma, T. (2018). Construction of an integrated database for herg blocking small molecules. *PLoS One* 13, e0199348

Wang, S., Sun, H., Liu, H., Li, D., Li, Y., and Hou, T. (2016). Admet evaluation in drug discovery. 16. predicting herg blockers by combining multiple pharmacophores and machine learning approaches. *Molecular pharmaceutics* 13, 2855–2866

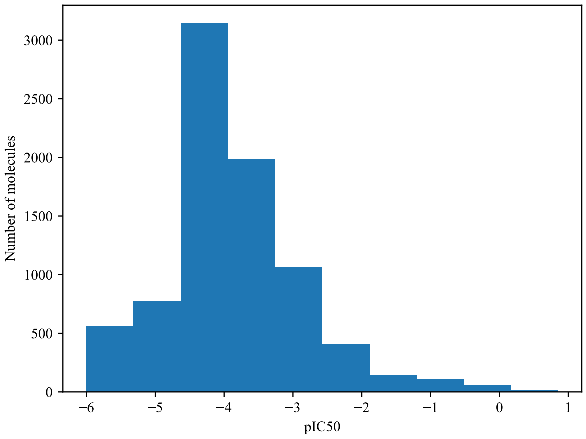

Figure S1. The distribution of pIC50 of the HERG-DB.

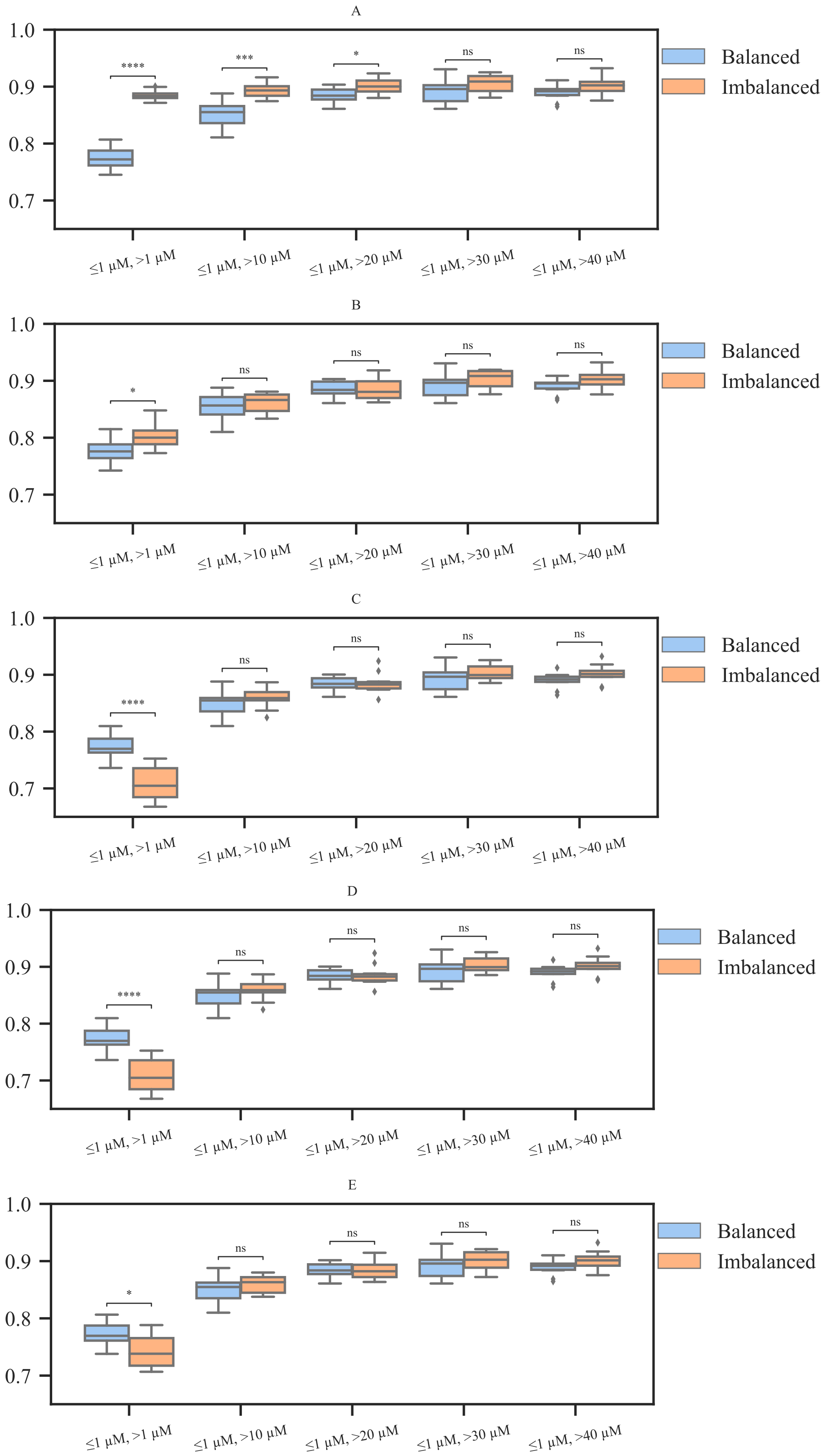

Note: ns (Not significant, P value ≥ 0.05), * (0.01 < P ≤ 0.05), ** (0.001 < P ≤ 0.01), *** (0.0001 < P ≤ 0.001), **** (0.00001 < P ≤ 0.0001)

Figure S2. The box plot of Accuracy between balanced and imbalanced dataset (A: Accuracy, B: Precision, C:Recall, D: AUC, E: F1-score)
